# From Biomaterial Innovation to Surgical Practice: A Systematic Review of RADA16 Self-Assembling Peptide Hydrogel in Otolaryngology and Head & Neck Surgery

**DOI:** 10.3390/jcm15062113

**Published:** 2026-03-10

**Authors:** Antonio Moffa, Domiziana Nardelli, Francesco Iafrati, Giannicola Iannella, Annalisa Pace, Peter Baptista, Manuele Casale

**Affiliations:** 1 School of Medicine, Università Campus Bio-Medico di Roma, Via Álvaro del Portillo 200, 00128 Rome, Italy; a.moffa@policlinicocampus.it (A.M.); f.iafrati@policlinicocampus.it (F.I.); m.casale@policlinicocampus.it (M.C.); 2 Unit of Integrated Therapies in Otolaryngology, Fondazione Policlinico Universitario Campus Bio-Medico, Via Álvaro del Portillo 21, 00128 Rome, Italy; 3 Department of ‘Organi di Senso’, University “Sapienza”, Viale dell’Università, 33, 00185 Rome, Italy; giannicola.iannella@uniroma1.it (G.I.); annalisa.pace@uniroma1.it (A.P.); 4 ENT Department, Al Zahra Private Hospital Dubai, Dubai 23614, United Arab Emirates; peterbap-tista@rocketmail.com

**Keywords:** RADA16 peptide, PuraBond^®^, PuraStat^®^, self-assembling peptides, haemostatics, hydrogels, otolaryngology, head and neck surgery, systematic review, postoperative haemorrhage, haemostasis, surgical, wound healing, tissue scaffolds, nanofibers, biomimetic materials

## Abstract

**Background**: Postoperative bleeding is a frequent complication in otolaryngology and head and neck surgery, often leading to readmissions and increased healthcare costs. **Objectives**: This systematic review evaluates the clinical efficacy, safety, and impact of RADA16, a synthetic self-assembling peptide hydrogel, as a topical haemostatic adjunct in this surgical field. **Methods**: In adherence with PRISMA 2020 guidelines, a systematic search of PubMed, Scopus, and Web of Science was conducted through December 2025. Eligible studies included adult patients undergoing otolaryngological or head and neck surgical procedures where RADA16 (CAS 289042-25-7, PuraBond^®^/PuraStat^®^/PuraGel^®^, ^®^, 3-D Matrix SAS; Caluire et Cuire, Lyon, France) was applied intraoperatively. Exclusion criteria included non-English publications, reviews, and studies without clinical outcome data. Risk of bias was assessed using the Cochrane Risk of Bias tool for RCTs and the Newcastle-Ottawa Scale for observational studies. A narrative synthesis was performed due to heterogeneity in outcome reporting. **Results**: Eight studies involving 1761 patients were included. In oropharyngeal surgery, RADA16 significantly reduced postoperative haemorrhage (6.3% vs. 16.7%, *p* = 0.016) and was associated with faster resumption of normal diet and lower pain scores (*p* = 0.016). In nasal surgery, it significantly lowered epistaxis rates (0.4% vs. 2.2%, adjusted OR 0.027, *p* = 0.026) and reduced the need for nasal packing. In cervical endocrine surgery, the rate of hematoma requiring revision was low (0.84%), with no delayed bleeding beyond 24 h. Surgeons consistently reported high satisfaction and ease of application. No serious device-related adverse events were reported. **Discussion**: Current evidence suggests RADA16 is a safe and effective haemostatic adjunct that can improve postoperative recovery and reduce readmission rates in specific surgical contexts. Limitations include heterogeneity in study designs, small sample sizes in some domains, and a lack of long-term follow-up. Further large-scale randomized controlled trials are needed to quantify its economic impact and formalize its role in surgical pathways. **Funding**: This study was funded by 3-D Matrix Medical Technology for article processing charges. The funder had no role in study design, data collection, analysis, interpretation, or writing. **Registration**: This review was not registered in a systematic review registry.

## 1. Introduction

Postoperative bleeding remains a persistent challenge in otolaryngology and head and neck surgery. The anatomical complexity, dense vascular networks, and proximity to critical aerodigestive structures predispose patients to haemorrhagic complications, which are the most common adverse event in both intraoperative and postoperative settings. Quantitatively, readmission rates attributable to postoperative haemorrhage reach 19% following adult tonsillectomy and up to 15% after inferior turbinate surgery. At the same time, the average cost of managing a single episode of epistaxis exceeds USD 400 [1,2]. These bleeding complications directly affect patient-centred outcomes, including increased pain, higher readmission rates, and prolonged hospital length of stay (LOS), collectively imposing a substantial clinical and economic burden on healthcare systems. Traditional haemostatic strategies, including nasal packing, electrocautery, and systemic antifibrinolytic agents such as tranexamic acid, are associated with notable limitations. While effective, nasal packing is often poorly tolerated, leading to patient discomfort, synechiae formation (i.e., abnormal adhesions between structures, such as the nasal septum and turbinates), and acting as a temporary foreign body. Furthermore, it carries a risk of septal perforation, with a recent analysis reporting a 5.3% incidence in patients managed for epistaxis in emergency settings [3]. A 2021 meta-analysis concluded that while systemic tranexamic acid can reduce intraoperative blood loss, it has not consistently demonstrated a significant reduction in secondary postoperative haemorrhage, particularly following tonsillectomy [4].

Current topical haemostatic agents used in otolaryngology include fibrin sealants such as Tisseel and Evicel, gelatin-thrombin matrices such as Floseal and Surgiflo, oxidized regenerated cellulose (e.g., Surgicel), chitosan-based haemostatic dressings, and topical antifibrinolytics such as tranexamic acid [5]. While effective, these agents present specific limitations. While fibrin sealants participate in the final stages of coagulation by supplying exogenous fibrinogen and thrombin, they rely on human or animal-derived components and need preparation before use [5]. Gelatin-thrombin matrices expand upon contact with blood and may exert a mass effect, which can be undesirable in confined head and neck anatomical spaces. Oxidized cellulose products act as mechanical scaffolds but may obscure the surgical field due to opacity [6]. Chitosan-based materials exhibit strong bioadhesion but can vary in handling characteristics [6]. Topical tranexamic acid acts by inhibiting fibrinolysis rather than by providing a structural haemostatic matrix [6].

Recent research in biomaterials has transitioned from traditional haemostatic gels toward biopolymer-based hydrogels and aerogels with tailored microstructures. These advanced scaffolds, such as composite kaolin/chitin aerogels, exhibit rapid in vitro coagulation and high water absorption, demonstrating the potential of engineered networks to optimize postoperative bleeding control [7,8]. In this context, the self-assembling peptide hydrogel RADA16 (RADARADARADARADA, CAS 289042-25-7, 3-D Matrix SAS; Caluire et Cuire, France) has emerged as a promising innovation. RADA16 is a synthetically engineered peptide composed of 16 amino acids [9]. Its sequence consists of regularly alternating arginine (R), alanine (A), and aspartic acid (D) residues, which introduce positive charge, hydrophobic character, and negative charge, respectively [10]. This repeating ionic pattern creates an amphiphilic structure, with charged residues on one face and hydrophobic side chains on the other. Upon exposure to physiological pH and ionic conditions, it rapidly self-assembles into a highly stable β-sheet conformation, resulting in a transparent, nanofibrous three-dimensional hydrogel matrix within seconds [9,11,12]. This matrix forms a conformable, biocompatible physical barrier that seals exposed microvasculature and mimics the native extracellular matrix (ECM), providing a scaffold that may promote epithelialization and favourable wound healing dynamics [13]. A 2025 meta-analysis of preclinical animal studies found that treatment with RADA16-based peptides resulted in a mean improvement in wound closure of 11.25% at 7 days and 9.48% at 14 days post-injury compared to controls [13].

The clinical utility of RADA16 is mainly documented in gastroenterology, where prospective studies have demonstrated its effectiveness in achieving endoscopic haemostasis across a range of bleeding aetiologies, including emergency and prophylactic settings, achieving immediate haemostasis in up to 94.2% of cases, including in patients on antithrombotic therapy [14,15,16,17]. Moreover, it was associated with reduced short-term rebleeding in selected cohorts. In addition to GI bleeding, RADA16 has been evaluated in other surgical specialties. A blinded randomized controlled trial (RCT) in laparoscopic gynaecologic surgery demonstrated 100% intraoperative haemostatic success with RADA16 compared to standard techniques [18]. Similarly, a feasibility study conducted in laparoscopic colorectal surgery settings reported safe and effective haemostasis in all 20 enrolled patients [19]. Emerging case-level evidence further suggests applicability in urology, including successful add-on haemostasis during holmium laser enucleation of the prostate, highlighting the agent’s adaptability to highly vascular operative fields [20].

Collectively, these findings support the safety, feasibility, and reproducible haemostatic performance of RADA16 hydrogels in this field, which involves thin mucosal surfaces, exposed neurovascular bundles, and limited operative corridors. Thermal-based haemostasis may induce protein denaturation and impair mucosal regeneration, potentially contributing to delayed bleeding and increased postoperative pain [21]. By contrast, RADA16 self-assembles into a transparent scaffold that mimics native ECM architecture and provides non-thermal mechanical haemostasis. Furthermore, its transparency permits continuous visualization of critical structures, such as the recurrent laryngeal nerve during thyroid surgery or exposed musculature in transoral resections. However, RADA16 represents a premium haemostatic adjunct, with acquisition costs that may exceed those of conventional cellulose or gelatine-based agents. Reimbursement policies vary internationally and may influence adoption across healthcare systems. Although the product is ready-to-use, optimal application requires a relatively dry field and familiarity with its self-assembly behaviour, introducing a modest learning curve. Currently, no systematic review or meta-analysis has synthesized the evidence for RADA16 within this field. Therefore, this systematic review aims to synthesize the current clinical evidence regarding RADA16 use in otolaryngology and head & neck surgery and to evaluate its impact on haemorrhage rates, hospital LOS, postoperative pain, and other patient-relevant outcomes.

## 2. Materials and Methods

The study was designed along the guidelines of the Centre for Review and Dissemination’s Guidance for Undertaking Review in Health Care, and is being reported in adherence with the Preferred Reporting Items for Systematic Review and Meta-Analyses (PRISMA) statement [22]. The completed PRISMA 2020 checklist (Table S1) is provided as a Supplementary file.

### 2.1. Data Source and Study Searching

A systematic review of the literature was performed in accordance with the PRISMA 2020 guidelines for systematic reviews [22]. Searches were conducted across Scopus (Elsevier, Amsterdam, The Netherlands), PubMed (National Library of Medicine, Bethesda, MD, USA), and Web of Science (Clarivate Analytics, Philadelphia, PA, USA) databases. The initial search was performed in December 2025 using the following Boolean phrase structured for PubMed and adapted as necessary for the syntax of each database: (“RADA16” OR “PuraBond^®^” OR “PuraStat^®^” OR “self-assembling peptide” OR “self-assembling peptide” OR “peptide hydrogel”) AND (“otolaryngology” OR “head and neck surgery” OR “tonsillectomy” OR “nasal surgery” OR “thyroidectomy” OR “parathyroidectomy” OR “TORS” OR “transoral robotic surgery”) AND (“haemostasis” OR “haemostasis” OR “haemostatic” OR “haemostatic” OR “bleeding” OR “haemorrhage” OR “haemorrhage” OR “postoperative bleeding”). A formal protocol was not registered (e.g., PROSPERO) prior to commencement. No language restrictions were applied during the initial search; however, only English-language publications were included in the final synthesis due to resource constraints for translation. Additionally, a manual cross-referencing search of the bibliographies of included studies and relevant review articles was performed by two independent investigators (A.M. and D.N.) to minimize the risk of missing relevant studies.

### 2.2. Inclusion/Exclusion Criteria

Study eligibility was determined using the Population, Intervention, Comparison, Outcome, and Study design (PICOS) framework [18]. The PICOS framework is a mnemonic used in Evidence-Based Medicine to frame and answer a clinical or health care-related question. It presents the following structure. P (Population): adult patients undergoing otolaryngological or head and neck surgical procedures. Given the diversity of procedures within this specialty, populations were analyzed in four subgroups: (1) oropharyngeal cancer surgery (including transoral robotic surgery), (2) tonsillectomy, (3) nasal surgery (e.g., turbinate resection, septoplasty, functional endoscopic sinus surgery), and (4) cervical endocrine surgery (e.g., thyroidectomy, parathyroidectomy). I (Intervention): intraoperative application of the self-assembling peptide hydrogel RADA16 (commercially available as PuraBond^®^, PuraGel^®^, or PuraStat^®^, 3-D Matrix SAS, Caluire-et-Cuire, France) as a topical haemostatic agent. C (Comparison): Control groups were categorized into three distinct comparator types: (a) No topical haemostatic agent: Patients received standard surgical care without any adjunctive topical haemostatic agent; (b) Alternative topical haemostatic agents: Patients received a different haemostatic agent (e.g., Tisseel^®^, (Baxter Healthcare Corp., Deerfield, IL, USA) Floseal^®^ (Baxter Healthcare Corporation)); (c) Standard haemostatic techniques: Patients received conventional haemostatic methods (e.g., diathermy, nasal packing) without a topical haemostatic agent. Studies employing these distinct comparator types were analyzed separately, and no pooled analysis was performed across different comparator categories. Direct comparisons between RADA16 and each comparator type are explicitly identified where applicable. O (Outcome): primary outcomes were postoperative haemorrhage rate, re-admission rate related to bleeding, and length of hospital stay, while secondary outcomes included time to resumption of normal diet, analgesia usage, pain scores, surgeon-reported ease of application and satisfaction, and device-related adverse events. S (Study design): RCTs, prospective and retrospective cohort studies, case series, and case reports were eligible for inclusion in our systematic review. Exclusion criteria included non-English publications, reviews, clinical guidelines, consensus statements, diagnostic studies, molecular/laboratory studies, observational studies, letters, and studies with unclear or incomplete data. Conference abstracts, trial registries, and gray literature were not included due to a lack of peer review and outcome detail. Studies not reporting on RADA16 or its commercial formulations were also excluded.

### 2.3. Data Extraction and Data Analysis

Two independent reviewers (A.M. and D.N.) performed the initial literature search and screening. Articles were first screened by title and abstract, followed by full-text assessment. Discrepancies were resolved through consensus or consultation with a third reviewer (F.I.). Data from eligible studies were extracted using a standardized form, including study characteristics (author, year, country, design, sample size), patient demographics, intervention details, comparator, outcome measures, and follow-up duration. Data extraction was verified independently by two reviewers (M.P. and E.D.N.). A meta-analysis was not feasible due to substantial clinical and methodological heterogeneity across the included studies, including (i) diverse surgical procedures and patient populations even within domains; (ii) variation in study designs (ranging from RCTs to case reports); (iii) inconsistent outcome definitions and reporting timeframes; and (iv) an insufficient number of comparable studies within any single domain to permit meaningful statistical pooling. Therefore, a narrative synthesis of findings was conducted, summarizing results by surgical domain: oropharyngeal cancer surgery, tonsillectomy, nasal surgery, and cervical endocrine surgery.

### 2.4. RADA 16 Composition and Application

RADA16 is a synthetic self-assembling peptide hydrogel with the amino acid sequence Ac-(RADA)_4_-CONH_2_, where R, A, and D represent arginine, alanine, and aspartic acid, respectively [5,6,7]. It is commercially available as PuraBond^®^, PuraGel^®^, and PuraStat^®^ (3-D Matrix SAS, Caluire-et-Cuire, France), supplied as a sterile, ready-to-use 2.5% (*w*/*v*) hydrogel in pre-filled syringes of 3 mL or 5 mL volumes [8]. The product requires no pre-operative preparation, thawing, or mixing, and can be stored refrigerated. Upon contact with physiological fluids or blood, the peptide monomers spontaneously self-assemble into a three-dimensional nanofibrous hydrogel matrix. This matrix adheres to moist tissue surfaces, forming a transparent physical barrier that promotes haemostasis through mechanical sealing and local concentration of clotting factors. The hydrogel is biodegradable and is deemed to have a low immunogenic potential, meaning it does not elicit a significant adaptive immune response, as it is composed of synthetic amino acids that degrade into natural byproducts [23]. The application of RADA16 varied by surgical procedure and study, as detailed below:

In transoral robotic surgery for oropharyngeal malignancies, 3 mL of PuraBond^®^ or 4 mL of PuraStat^®^ (one syringe) were typically applied to the surgical bed following resection and achievement of haemostasis [24,25]. The gel was delivered via a long-tipped applicator for unobstructed access. Surgeons emphasized the importance of a dry surgical field before application. No subsequent disruption of the gel was recommended. In adult tonsillectomy, a single 5 mL syringe was used for both tonsillar fossae [2]. After haemostasis and wound bed cleaning, RADA16 was applied as a thin, wide layer covering approximately 2 cm × 2 cm per tonsil bed, with 0.5–1.0 mL used per side. Repeat application to bleeding sites was permitted at the surgeon’s discretion. In nasal procedures such as inferior turbinate resection, 1 mL of 2.5% RADA-16 was applied along the cut turbinate surface and spread using a Goldman elevator [26]. The gel was allowed to adhere for at least 30 s before excess was suctioned. The mean total dose reported in one multicentre series was 3.12 mL for bilateral nasal application [27]. In thyroid and parathyroid procedures, 3 mL of PuraStat^®^ was applied intraoperatively via a thin application nozzle directly to the surgical bed, often in contact with critical structures such as the recurrent laryngeal nerves. In salvage surgery for post-radiotherapy stenosis, 4 mL of PuraStat^®^ was applied to raw mucosal surfaces after confirmation of haemostasis, following corticosteroid injection [28].

### 2.5. Risk of Bias Assessment

Risk of bias was assessed independently by two reviewers (D.N. and F.I.) using tools appropriate to each study design. For the RCT, the Cochrane Risk of Bias tool for randomized trials (RoB 2.0) was used [29]. For non-randomized studies (cohort studies and case series with comparators), the Risk Of Bias In Non-randomized Studies—of Interventions (ROBINS-I) tool [30] was applied. Single-arm case series and case reports were not formally assessed using ROBINS-I due to the inherent lack of a comparator group; these were noted as providing very low certainty evidence by design. Disagreements were resolved through consensus or consultation with a third reviewer (A.M.).

### 2.6. Certainty of Evidence Assessment

The certainty of the body of evidence for primary outcomes (postoperative haemorrhage rate, readmission rate, and length of stay) was assessed using the Grading of Recommendations, Assessment, Development, and Evaluations (GRADE) framework [31]. Two reviewers (D.N. and F.I.) independently evaluated each outcome across the four surgical domains, considering risk of bias (informed by RoB 2.0 and ROBINS-I assessments), inconsistency, indirectness, imprecision, and publication bias. Evidence was categorized into four levels: high, moderate, low, or very low certainty. Disagreements were resolved through consensus or consultation with a third reviewer (A.M.).

## 3. Results

**Figure 1:**
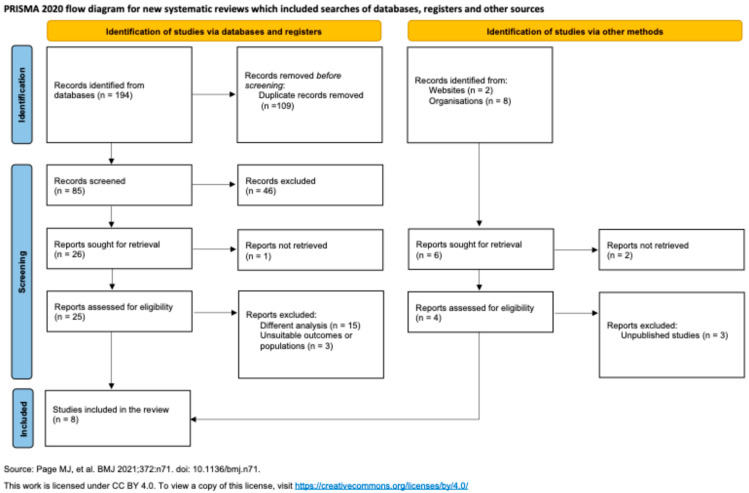
PRISMA 2020 flow diagram for systematic reviews [22]. Initial identification yielded 194 records from databases and 10 from other methods. After 109 duplicates were removed, 85 records were screened, and 46 were excluded. Of the remaining reports sought for retrieval, 25 database reports and 4 additional reports were assessed for eligibility. Reports were excluded for different analyses (*n* = 15), unsuitable outcomes or populations (*n* = 3), or being unpublished studies (*n* = 3). Ultimately, 8 studies were included in the review.A total of 1761 patients were included in the analysis, with a mean age of 48.2 ± 15 years. Baseline characteristics of the study populations are illustrated in Table 1. Procedures were analyzed across four distinct surgical domains, as illustrated in Table 2: oropharyngeal cancer surgery (*n* = 99 procedures), tonsillectomy (*n* = 21), nasal surgery (*n* = 738), and cervical endocrine surgery (*n* = 353). An additional 903 procedures from comparative groups are included in the analysis. The synthesized findings are presented below in Table 1 and Table 2.

A systematic search of the literature yielded 204 articles for initial screening. After the removal of 178 duplicates, 26 full-text articles were assessed for eligibility. Of these, 18 were excluded for not meeting the inclusion criteria, resulting in 8 studies selected for final analysis, as illustrated in Figure 1.

**Table 1:** Baseline characteristics of the included studies.

Oropharyngeal Cancer									
Author (Year)	Country	Study Design	*N.* of Patients	Sex (M/F)	Age (Mean ± SD)	Indication	Control	RADA16 Formulation (Dose)	Follow-Up
Wong et al. 2020 [24]	Australia	Case Report	*n* = 1	1/0	49	Post-radiotherapy nasopharyngeal/palatal stenosis for HPV+ tongue base SCC	None	4 mL of PuraStat^®^ to the surgical bed	2 months
Gupta et al. 2022 [25]	United Kingdom	Case series study	*n* = 12	6/6	55	TORS for HPV+ OPSCC (lateral oropharyngectomy, partial oropharyngeal resection, oropharyngeal re-resection)	None	3 mL of PuraBond^®^ to the surgical bed	4 weeks
Ghazal et al. 2025 [32]	United Kingdom	Blinded RCT	*n* = 68 (PuraBond^®^ = 32; Control = 36)	51/17	64	Trans-oral resection of oropharyngeal/oral cavity neoplasias	No topical haemostatic agent	PuraBond^®^ application to the surgical bed	30 days
Sattar et al. 2025 [33]	United Kingdom	Retrospective cohort study	*n* = 18 (PuraBond^®^ = 6; Control = 12)	Not reported	55 ± 18.9	TORS (tonsillectomy; tongue base mucosectomy; lateral oropharyngectomy; selective II-IV neck dissection; ECA ligation or combinations of the latter)	Tisseel (*n* = 8) or Floseal (*n* = 4)	PuraBond^®^/Tisseel (4 mL)/Floseal application to the surgical bed	30 days
**Tonsillectomy**									
Michaels et al. 2024 [2]	United Kingdom	Prospective case series and historical control group	*n* = 184 (PuraBond^®^ = 21; Control (no topical haemostasis) = 164)	56/129 (PuraBond^®^ = 3/18; Control = 53/111)	27.7 ± 6.2 (PuraBond^®^ = 23.3; Control = 32)	Bilateral tonsillectomy for tonsillitis (*n* = 118), histological characterization (*n* = 58); quinsy (*n* = 7); obstructive sleep apnea (*n* = 2)	No topical haemostatic agent	5 mL PuraBond^®^ tube shared between both surgical beds (2 cm × 2 cm)	No follow-ups were routinely scheduled
**Nasal Surgery**									
Xu et al. 2025 [26]	USA	Retrospective study	*n* = 958 (PuraBond^®^ = 571; Control = 414)	509/476 (PuraBond^®^ = 201/213; Control = 308/263)	PuraBond^®^ = 43.0 ± 19.1; Control = 37.9 ± 16.7	Inferior turbinate resection	No topical haemostatic agent	1 m of PuraGel^®^ along the cut surface of each turbinate	>3 weeks
Soodin et al. 2022 [27]	Australia	Prospective multicentre case series	*n* = 167	Not reported	Not reported	Inferior turbinate reduction surgery ± FESS (maxillary antrostomy, ethmoidectomy) ± septoplasty	No topical haemostatic agent	Mean dose: 3.12 mL of PuraStat^®^ for both sides of the nose.	2–5 weeks
**Cervical endocrine surgery**									
Gangner et al. 2022 [28]	France	Real-life single-centre retrospective case series	*n* = 353	72/281	54 ± 14.1	Primary surgery: 342; Completion surgery: 15 (lobo-isthmectomy; total thyroidectomy ± cervical node dissection ± parathyroidectomy; thyroglossal cyst)	No topical haemostatic agent	3 mL of PuraStat^®^ to the surgical bed	6.16 weeks

**Table 2:** Study results. (**a**) Primary outcomes, (**b**) Secondary outcomes.

(**a**)			
**Oropharyngeal Cancer**			
**Author (Year)**	**Post-Operative Haemorrhage Rate**	**Re-Admission Rate**	**LOS (days)**
Wong et al. 2020 [24]	None	None	Patients discharged on same day (0).
Gupta et al. 2022 [25]	No patients who developed, either a primary or secondary, haemorrhage post-TORS (*n* = 0%)	None	Mean LOS: 2.87 ± 0.93
Ghazal et al. 2025 [32]	PuraBond^®^ = 6.3% (*n* = 2); Control = 16.7% (*n* = 6)	None	Mean LOS: PuraBond^®^ = 2.7; Control = 3.0
Sattar et al. 2025 [33]	*n* = 5.6% (*n* = 1, PuraBond^®^ group), managed conservatively	*n* = 11.1% (*n* = 2: 1 PuraBond^®^ for haemorrhage, 1 Tisseel for pain)	Mean LOS: 3.7 ± 0.8 (one outlier of 26.0 days due to pneumonia)
**Tonsillectomy**			
Michaels et al. 2024 [2]	PuraBond^®^ = 4.8% (*n* = 1/21); Control = 14.6%, (*n* = 24/164)	PuraBond^®^ = 9.5%, (*n* = 2/21); Control = 18.9% (*n* = 1/164); *p* = 0.378. Main reasons were haemorrhage (*n* = 24 controls, *n* = 1 PuraBond^®^). 2 control patients needed re-operation (0% RADA16). Remaining re-admissions were due to pain	All RADA16 subjects had day-case surgery (0), whereas 7.9% of controls were overnight admissions (1).
**Nasal surgery**			
Xu et al. 2025 [26]	PuraBond^®^ *n* = 0.4% (*n* = 2), Control 2.2% (*n* = 9), OR adjusted for coagulation 0.027.	Not reported	Not reported
Soodin et al. 2022 [27]	14 reported minor self-resolving bleeding (3 were on anti-coagulant medication and one had a Von Willebrand Disease); 5 patients reported bleeding (3 early, 2 late) needing additional treatment. All cases were treated locally with nasal packing/tranexamic acid. None returned to operating room.	None	Not reported
**Cervical endocrine surgery**			
Gangner et al. 2022 [28]	Hematoma requiring revision surgery 0.8% (*n* = 3), no delayed bleeding after 24 h	4 (1.12%) for haematoma/suspected haematoma	All patients were discharged the day after surgery (1).
(**b**)			
**Oropharyngeal Cancer**			
**Author (Year)**	**Post-Surgical Adverse Events**	**Surgeon-Reported Ease of Application**	**Other Outcomes**
Wong et al. 2020 [24]	None	Not reported	Not reported
Gupta et al. 2022 [25]	None	Easy application on all procedures (100%)	No patient required any type of feeding tube or a tracheostomy either prior to, during or within 30 days of TORS (*n* = 0%).
Ghazal et al. 2025 [32]	Cerebral infarct: Control (*n* = 1); Wound infection: PuraBond^®^ (*n* = 2) Post-operative infection: PuraBond^®^ (*n* = 3) Control (*n* = 1); First bite syndrome: Control (*n* = 1); Postoperative urinary retention: Control (*n* = 1). Bleeds in PuraBond^®^ occurred mostly at 10–11 days post-op, in days 2–10 in controls.	Easy application in 89% of cases (surgeons reported need for a longer applicator).	Mean time to full normalcy of oral diet PuraBond^®^ = 1.2 ± 9.1; Control = 17.0 ± 9.5 (*p* = 0.79); Aveage pain scores: Days 1–7: PuraBond^®^ = 3.3 ± 2.8; Control = 4.4 ± 2.9 (*p* = 0.098) Days 1–14: PuraBond^®^ = 3.0 ± 2.7; Control = 3.9 ± 2.9 (*p* = 0.011) Days 1–30: PuraBond^®^ = 2.6 ± 2.6; Control = 3.4 ± 3.0 (*p* = 0.016). Post-op opioid usage at day 1 post-op: 34.4% PuraBond^®^, 58.3% controls (*p* > 0.05).
Sattar et al. 2025 [33]	Death secondary to post-operative pneumonia (*n* = 1).	Easy and surgeon friendly application	All patients resumed oral intake on day 1 post-operatively.
**Tonsillectomy**			
Michaels et al. 2024 [2]	Haemorrhage (see re-admission rate)	Handling/delivery: 4.8 ± 0.5/5; Haemostatic effectiveness: 4.3 ± 1/5; Transparency benefits: 4.3 ± 0.5/5; Ready-to-use format: 4.5 ± 0.6/5; Overall satisfaction: 4.3 ± 0.5/5	Not reported
**Nasal surgery**			
Xu et al. 2025 [26]	Not reported	Not reported	Average cost associated with epistaxis (per patient): PuraBond^®^ USD 379.7, Control USD 436.2
Soodin et al. 2022 [27]	Bleeding (*n* = 19); Crusting (*n* = 38); Adhesions (*n* = 14); Infection (*n* = 4)	Handling/delivery: 4.6/5; Haemostatic effectiveness: 4.4/5; Transparency benefits: 4.7/5; Ready-to-use format: 4.7/5; Overall satisfaction: 4.8/5	Rate of adhesions in areas where PuraBond^®^ was applied, and which required treatment was 4.2% (7/167). Noticeable crusting in 17 patients (10.2%). 5 cases of minor adhesions, 9 cases of significant adhesions, of which 2 were excluded from the analysis. as they did not develop in areas where PuraStat^®^ has been applied (4.2%). 4 cases of infection.
**Cervical endocrine surgery**			
Gangner et al. 2022 [28]	None	Not reported	Dysphonia at 6 weeks post-op (*n* = 15); Hypocalcemia < 24 h (*n* = 4); Tingling to fingers at 6 weeks post-op (*n* = 8); Lack of response to electrical stimulation of laryngeal nerves stimulation at end of surgery (*n* = 12)

### 3.1. Oropharyngeal Cancer Surgery

The most robust evidence comes from the single-centre, blinded, RCT by Ghazal et al. (*n* = 68) [32]. This study investigated RADA16 (PuraBond^®^, 3-D Matrix SAS; Caluire et Cuire, France) in the resection of dysplasia or malignancy of the oral cavity/oropharynx, with patients randomized 1:1 and stratified for lesion type, surgical technique, and neck dissection. While no primary haemorrhages occurred in either group, the RADA16 arm (*n* = 32) demonstrated a significantly lower postoperative haemorrhage rate compared to the control group, which received no topical haemostasis (6.3% vs. 16.7%, *p* = 0.016). Notably, bleeds in the experimental group occurred later (postoperative days 10 and 11) compared to the control group (days 2–10). Furthermore, RADA16 use was associated with a shorter mean length of stay (LOS: 2.7 vs. 3.0 days), faster resumption of a normal diet (1.2 vs. 17 days), reduced analgesic use (including opiates), and statistically significant drops in pain scores over 1–30 days (*p* = 0.016). When surveying surgeons, ease of application was reported as easy to apply in 89% of cases.

In the specific context of transoral robotic surgery (TORS, with da Vinci^®^ surgical robot, Intuitive Surgical Inc., Sunnyvale, CA, USA) for oropharyngeal cancer, findings from case series support its haemostatic efficacy. Gupta et al. [25] applied RADA16 (PuraBond^®^) in 13 TORS procedures for patients affected by Human Papilloma Virus-positive oropharyngeal squamous cell carcinoma and reported no primary or secondary postoperative haemorrhages. There were also no re-admissions within 30 days, with a mean LOS of 2.9 ± 0.9 days. Surgeons rated the application as “easy” in 100% of cases, with no complications at follow-up. This aligns with the findings of Sattar et al. [33] in a retrospective cohort of 18 patients who underwent TORS. The most common indications were suspected tonsillar primary cancer (*n* = 7), carcinoma of unknown primary (*n* = 5), and recurrent tonsillitis (*n* = 5). 12 cases (66.7%) involved a concurrent procedure, most commonly external carotid artery ligation, panendoscopy, or neck dissection. PuraBond^®^ was applied in 6 cases, while the remaining patients received Tisseel (*n* = 8) or Floseal (*n* = 4). In this complex setting, one patient (5.6%) from the RADA16 group experienced a secondary haemorrhage, which was managed conservatively. Finally, in a complex salvage case reported by Wong et al. [24] involving the division of a post-radiotherapy nasopharyngeal/palatal stenosis, the application of RADA16 (PuraBond^®^) resulted in no postoperative haemorrhage, a same-day discharge (LOS 0 days), no readmission, and an absence of complications at one-month follow-up.

### 3.2. Tonsillectomy

Evidence in this domain is limited to one prospective case series. Michaels et al. [2], RADA16 (PuraBond^®^) investigated the efficacy of RADA16 in the context of adult tonsillectomy. The study included 21 patients in whom RADA16 was applied in a thin layer to the tonsillar fossae following diathermy. These were compared with 164 historical controls undergoing the same procedure without the agent. The authors reported a 67% relative reduction in readmissions specifically for postoperative haemorrhage (*p* = 0.317) and a 50% reduction in all-cause readmission (*p* = 0.378). No patient in the RADA16 group required a return to the operating theatre for persistent bleeding, compared to two control patients (1.2%). Intra-operative assessment by four surgeons indicated high overall satisfaction (4.25/5), particularly regarding ease of use, haemostatic efficacy, and gel transparency. No device-related adverse events were recorded, although no routine follow-up protocol was scheduled.

### 3.3. Nasal Surgery

Evidence in this domain is limited to two prospective studies. Xu et al. [26] conducted a large retrospective analysis of 985 patients undergoing inferior turbinate resection, comparing 571 who received RADA16 (PuraStat^®^, 3-D Matrix SAS; Caluire et Cuire, France) to 414 who did not. The overall postoperative epistaxis rate was significantly lower in the PuraStat^®^ cohort (*p* < 0.05). After adjusting for confounders, including a higher rate of anticoagulant use in the control group, the PuraStat^®^ application was associated with a significantly lower risk of postoperative epistaxis (*p* = 0.026). A cost-effectiveness analysis indicated a lower mean bleeding-related cost per patient with PuraStat^®^ (USD 379.74 vs. USD 436.21), though this difference was not statistically significant (*p* = 0.36). These findings are supported by the prospective multicentre case series by Soodin et al. [27] (*n* = 167 procedures including turbinate reduction ± septoplasty/FESS). With a mean application of 3.12 mL per patient, PuraStat^®^ achieved a haemostatic efficacy of 98.2%. This allowed 78% of procedures to be performed without nasal packing and with minimal diathermy (86%). Only 2.9% of patients required additional treatment for postoperative bleeding, with no returns to theatre. The agent was associated with minimal crusting, a low rate of treatment-requiring adhesions (4.2%), and no device-related adverse events. Surgeon satisfaction was very high, with an overall score of 4.8/5, particularly for ease of use, transparency, and haemostatic effectiveness.

### 3.4. Cervical Endocrine Surgery

Evidence in this domain is limited to one prospective study. The efficacy and safety of RADA16 (PuraStat^®^) in open cervical endocrine surgery were evaluated by Gangner et al. [28] in a large, retrospective real-life case series encompassing 353 procedures (336 thyroidectomies and 21 parathyroidectomies). PuraStat^®^ was applied intra-operatively as the sole topical haemostatic agent, frequently in direct contact with critical structures such as the recurrent laryngeal nerves and parathyroid glands. Postoperatively, only 3 of 357 procedures (0.84%) required revision surgery for a cervical hematoma, with all events occurring within the first 4 h and no delayed bleeding beyond 24 h. At a mean follow-up of 6.2 weeks, no device-related adverse events were reported.

### 3.5. Risk of Bias Assessment

**Figure 2:**
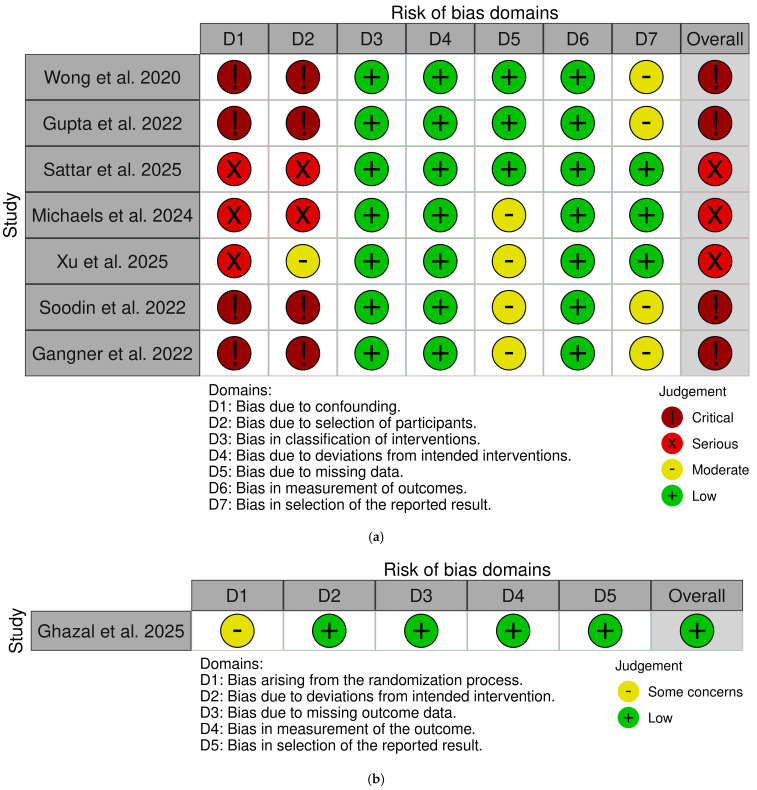
Risk of bias assessment [2,24,25,26,27,28,32,33]. (**a**) Risk of bias assessment for non-randomized studies using the ROBINS-I tool. (**b**) Risk of bias assessment for randomized trials using the RoB tool.

The RCT by Ghazal et al. [32] was judged to have low/some concerns overall due to incomplete reporting of random sequence generation and allocation concealment. All other domains (deviations from intended interventions, missing outcome data, outcome measurement, and selective reporting) were judged as low risk. Among non-randomized studies, the two retrospective cohort studies [1,33] and the prospective case series with historical control [2] were rated as having a serious risk of bias. This was primarily due to potential confounding, selection bias, and lack of adjustment for baseline differences between groups. The single-arm case series [25,27,28] and the case report [24] were rated as having a critical risk of bias due to the absence of comparator groups, which precludes adjustment for confounding and limits causal inference. The risk of bias for the included studies is illustrated in Figure 2a,b with the use of the Risk-of-bias VISualization (robvis) tool (Version: 0.3.0) [34].

### 3.6. Certainty of Evidence (GRADE) Assessment

In the domain of oropharyngeal cancer surgery, the overall certainty of evidence for a decrease in postoperative haemorrhage, bleeding-related readmission, and length of stay was judged as moderate. This rating was primarily supported by one RCT, while being downgraded for some concerns regarding risk of bias and for imprecision related to sample size. In adult tonsillectomy, the certainty of evidence was judged as very low, as conclusions were derived from a single prospective case series with historical controls. Serious risk of bias, lack of randomization, small intervention sample size, and imprecision significantly limited confidence in the estimated effects. For nasal surgery, the certainty of evidence was rated as low. Although one large retrospective comparative study demonstrated a statistically significant reduction in postoperative epistaxis, the observational design and potential confounding resulted in a downgrade for risk of bias. In cervical endocrine surgery, evidence was graded as very low, as findings were based solely on a single-arm retrospective case series without a comparator group. While the observed rate of hematoma requiring revision surgery was low, the absence of a control arm precludes causal inference. Formal assessment of publication bias using funnel plots was not feasible due to substantial clinical heterogeneity across surgical domains, differences in study design, and inconsistent outcome reporting. The limited number of studies within each domain further precluded meaningful pooled analysis (*n* < 10). Nevertheless, potential publication bias cannot be excluded. Given the commercial availability of RADA16 and industry involvement in some studies, selective reporting of positive outcomes is possible. Additionally, the exclusion of non-English studies and conference abstracts may have contributed to an overestimation of treatment effects.

## 4. Discussion

The strongest evidence derives from oropharyngeal cancer surgery, where a blinded RCT (*n* = 68) demonstrated significant reductions in postoperative haemorrhage (6.3% vs. 16.7%, *p* = 0.016), earlier diet resumption (1.2 vs. 17 days), lower pain scores (*p* = 0.016), and reduced analgesia requirements with RADA16 versus no topical haemostasis [32]. Supporting case series in TORS reported no haemorrhage events and high surgeon satisfaction [25,33]. In nasal surgery, a large retrospective cohort (*n* = 985) found significantly lower epistaxis rates with RADA16 (0.4% vs. 2.2%, *p* = 0.026) and reduced bleeding-related costs [26,27]. A prospective case series (*n* = 167) reported 98.2% haemostatic efficacy, enabling 78% of procedures to be performed without nasal packing [24]. In tonsillectomy, a prospective series with historical control (*n* = 184) observed 67% relative reduction in haemorrhage-related readmissions and 100% day-case discharge with RADA16 versus 92.1% in controls, though not statistically significant [2]. In cervical endocrine surgery (*n* = 353), hematoma requiring revision occurred in only 0.84% of cases, with no delayed bleeding beyond 24 h [28]. These findings align with data from gastrointestinal endoscopy, where RADA16 achieves immediate haemostasis in 94–100% of patients, including those on antithrombotic therapy [14,16,17]. In laparoscopic gynaecologic surgery, a blinded RCT reported 100% haemostatic success versus standard techniques [18]. Similar efficacy has been demonstrated in laparoscopic colorectal surgery [15] and urology [16]. Importantly, patient-centered outcomes remain underreported. While reductions in postoperative pain and earlier diet resumption were observed in oropharyngeal surgery, validated measures of swallowing function, quality of life, nasal airflow, and return to normal diet were not consistently assessed across studies [32,33]. Given the functional relevance of mucosal integrity in ENT surgery, future trials should incorporate standardized patient-reported outcome measures (PROMs) to better capture clinically meaningful benefit. Across the reviewed studies, the most consistently reported PROMS associated with RADA16 use include significant reductions in postoperative pain scores and a substantially faster resumption of a normal oral diet. 

The observed reductions in pain and earlier diet resumption [32,33] may be attributable to RADA16’s biomimetic properties. Upon exposure to physiological ionic strength and pH, it undergoes a rapid phase transition, shifting from a viscous solution to a stable, nanofibrous β-sheet matrix. This process creates a conformable mechanical barrier that seals microvasculature without the need for thermal energy, which can otherwise induce protein denaturation and delay mucosal healing [11,24]. The hydrogel also modulates inflammation by sequestering DAMPs and reducing TNF-α/IL-6 [35]. Furthermore, RADA16 undergoes slow biodegradation to amino acids without immunogenicity [23], explaining the absence of device-related adverse events across all included studies. The hydrogel is supplied in pre-filled, ready-to-use syringes (3 mL or 5 mL), requiring no preparation, thawing, or mixing, offering a practical advantage in the operating theatre over agents like fibrin sealants [20]. The standard application involves achieving initial haemostasis and a dry field, followed by direct delivery to the wound bed via a syringe with a long-tip or thin nozzle applicator [20,21,22]. The gel is allowed to self-assemble and adhere for approximately 30 s before excess is suctioned [23,24]. Dosing is procedure-specific, ranging from 1.0 mL per nasal fossa in inferior turbinate resection [26] to 4 mL in the surgical bed for OSCC resection [24]. Upon contact with fluids, the peptide monomers rapidly self-assemble into a transparent hydrogel matrix that adheres to tissue surfaces, forming a conformable physical barrier [5,6,7]. Its intrinsic transparency allows continuous visualization of underlying structures during application, which may be particularly relevant in anatomically constrained fields such as thyroid surgery near the recurrent laryngeal nerve [25]. From a mechanistic perspective, RADA16 may offer procedure-specific advantages in ENT surgery. Unlike electrocautery or thermal coagulation devices, which achieve haemostasis through tissue desiccation and thermal protein denaturation, RADA16 provides non-thermal mechanical haemostasis. This may reduce collateral mucosal injury, preserve microvascular perfusion, and potentially limit postoperative crusting or synechiae formation, particularly in nasal surgery [21]. Additionally, the ECM-mimicking nanofibrous scaffold may support more organized epithelial migration and mucosal healing kinetics, although this remains to be confirmed in procedure-specific clinical studies [23,35,36]. A 2025 meta-analysis of preclinical animal studies found that treatment with RADA16 resulted in a mean improvement in wound closure of 11.25% at 7 days and 9.48% at 14 days post-injury compared to controls [26]. This evidence suggests that RADA16 may facilitate not only haemostasis but also a more organized and accelerated tissue repair, which could explain the observed reductions in postoperative pain and earlier resumption of oral intake noted in some included studies [19].

Available evidence suggests that RADA16 is best positioned as an add-on for controlling diffuse capillary or venous oozing following primary haemostasis, rather than as a stand-alone solution for active arterial bleeding [8]. In settings of high-flow haemorrhage, space-occupying or vessel-specific haemostatic strategies remain necessary. Recognizing this distinction is essential to optimizing patient selection and surgical outcomes. Interestingly, surgeons surveyed in one included study indicated the need for a longer applicator [32]. Evidence regarding safety in anticoagulated patients remains limited. Among the included studies, only Xu et al. stratified outcomes upon anticoagulant use in a large retrospective cohort of nasal surgery patients [26]. In this subgroup analysis, postoperative bleeding occurred in 0.4% of patients treated with PuraBond^®^ (*n* = 2) compared with 2.2% in controls (*n* = 9), with an adjusted odds ratio for bleeding of 0.027 after controlling for coagulation status. While these findings suggest potential benefit in anticoagulated patients, the observational design precludes causal inference. Therefore, dedicated prospective trials are required before firm recommendations can be made. No study specifically evaluated patients with inherited coagulopathies or those undergoing salvage surgery after radiotherapy, limiting extrapolation to these high-risk populations. In terms of economic impact, the acquisition cost for RADA16, reported as approximately £200–250 per 3 mL syringe, represents a direct expenditure that must be evaluated against potential indirect savings [20]. These may include costs averted through reduced rates of postoperative bleeding, emergency department visits, unplanned readmissions, and the need for adjunctive packing [1].

This systematic review has several limitations that must be considered when interpreting the findings. The included studies span the entire evidence hierarchy, from a single RCT [21] to retrospective cohort studies [1,22], prospective and retrospective case series [2,20,23,24,25], and a case report [26]. This heterogeneity precluded meta-analysis and limits the strength of causal inferences that can be drawn. While the RCT provides level I evidence for oropharyngeal cancer surgery, evidence for other domains relies predominantly on observational studies with inherent susceptibility to confounding and selection bias. However, case reports and small case series were included, given the emerging nature of RADA16 use in otolaryngology and the limited number of RCTs available. While such designs inherently provide low-certainty evidence, their inclusion allowed a more comprehensive mapping of early clinical experience across different surgical domains. Moreover, the majority of included studies were rated as having a serious or critical risk of bias. Sample sizes were small in several domains (e.g., tonsillectomy *n* = 21 intervention), yielding imprecise effect estimates with wide confidence intervals. Outcome definitions and follow-up periods were inconsistent, ranging from 24 h to 6 weeks, preventing assessment of delayed complications. Important confounders, including bleeding risk factors and comorbidities, were incompletely reported, and generalizability is limited to four surgical domains in high-income countries. Review methodology limitations include exclusion of gray literature, lack of PROSPERO registration, and inability to perform meta-analysis. Additionally, although no language restrictions were applied during database searching, only English-language studies were included in the final synthesis. This may introduce language bias and potentially limit the completeness of the evidence base.

However, the review was conducted in accordance with PRISMA 2020 guidelines, and predefined eligibility criteria were applied consistently during study selection. Finally, this review received industry funding for article processing charges, though the funder had no role in study conduct or interpretation.

Current evidence suggests that RADA16 may represent a safe and potentially effective haemostatic adjunct in selected otolaryngologic procedures. However, the strength of this conclusion is limited by the predominance of observational data and small sample sizes across several domains. Its potential to provide a biocompatible scaffold that may favourably influence wound healing represents a significant advance beyond purely mechanical haemostatic agents. However, its application should be guided by an understanding of its specific indication for capillary bleeding. Future research should prioritize multicenter, adequately powered randomized controlled trials with standardized bleeding definitions and prespecified primary endpoints. Head-to-head comparisons with established haemostatic agents (e.g., fibrin sealants or oxidized cellulose) are essential to determine relative effectiveness and cost–benefit profiles. Long-term follow-up assessing mucosal healing, synechiae formation, and functional recovery should be incorporated. Additionally, formal cost-utility analyses and subgroup analyses in anticoagulated or high-risk patients are needed to define optimal patient selection criteria.

## 5. Conclusions

Evidence from 1761 patients across eight studies indicates that RADA16 self-assembling peptide hydrogel is a safe and effective topical haemostatic adjunct in otolaryngology and head and neck surgery. Clinical data demonstrate that its application significantly reduces postoperative haemorrhage rates in oropharyngeal and nasal procedures. Beyond its primary haemostatic function, RADA16 offers clinical and practical advantages, including lower pain scores, a faster return to a normal diet for oropharyngeal cancer patients, and a reduced need for nasal packing. Its transparency further aids surgeons by allowing continuous visualization of critical anatomical structures during application. Surgeons consistently report high satisfaction due to the product’s ready-to-use format and ease of application, even in the anatomically confined spaces characteristic of TORS and nasal surgery. While these results are promising, evidence is currently limited by study heterogeneity and a lack of long-term follow-up. Future large-scale randomized controlled trials and head-to-head comparisons with established haemostatic agents are essential to formalize its role in clinical pathways and further quantify its economic impact.

## Data Availability

No new data were created or analyzed in this study. Data sharing does not apply to this article.

## Supplementary Materials

The following supporting information can be downloaded at: https://www.mdpi.com/article/10.3390/jcm15062113/s1, Table S1: PRISMA 2020 checklist [37].

## Abbreviations

The following abbreviations are used in this manuscript:

GI	Gastrointestinal
LOS	Length of stay
PICOS	Population, Intervention, Comparison, Outcome, Study design
PRISMA	Preferred Reporting Items for Systematic Review and Meta-Analyses
RADA16	Self-assembling peptide hydrogel (commercially available as PuraBond^®^/PuraStat^®^/PuraGel^®^t)
RCT	Randomized Controlled Trial
TORS	Transoral Robotic Surgery
